# Factors Influencing the Severity of Acute Radiation-Induced Skin and Mucosal Toxicity in Head and Neck Cancer

**DOI:** 10.7759/cureus.18147

**Published:** 2021-09-21

**Authors:** Robin Chugh, Yashwant s Bisht, Vipul Nautiyal, Rashmi Jindal

**Affiliations:** 1 Dermatology, Himalayan Institute of Medical Sciences, Dehradun, IND; 2 Radiotherapy, Himalayan Institute of Medical Sciences, Dehradun, IND

**Keywords:** skin and mucosal toxicity, skin, severe, acute, dermatitis, radiation

## Abstract

Background and purpose

Radiotherapy is a crucial part of cancer therapy armamentarium, but it is associated with skin and mucosal toxicity in a substantial proportion of patients with head and neck cancer. Its extent, however, depends on several patient-related and treatment-related factors. In-depth knowledge of these is prudent for better patient management.

Aim

The aim of this study is to assess the factors influencing the severity of acute radiation-induced skin and mucosal toxicity in patients with head and neck cancer receiving external beam radiotherapy.

Materials and methods

This longitudinal observational study included all patients receiving curative external beam radiotherapy for head and neck cancer aged 18 years or above from January 2018 to December 2018. Patient-related and treatment-related characteristics including age, gender, type, staging and site of cancer, history of smoking and diabetes, surface area exposed, and concurrent chemotherapy were compared in patients experiencing severe and non-severe acute skin and mucosal toxicity using the Radiation Therapy Oncology Group (RTOG) scoring system.

Results

Higher age (p = 0.002), TNM stage IV (p = 0.023), and concurrent administration of chemotherapy (p = 0.002) were statistically associated with severe acute radiation-induced skin and mucosa toxicity, whereas gender, surface area irradiated, history of smoking, and diabetes did not show such an association.

Conclusion

Older patients with TNM stage IV malignancy receiving concurrent chemotherapy are at a high risk of developing skin and mucosal toxicity that might interfere with the treatment protocol and warrant hospitalization, compromising their quality of life.

## Introduction

Radiotherapy forms an essential component of the cancer therapy armamentarium. It is curative in a vast subset of head and neck cancers and, if indicated, can be combined with surgery and chemotherapy. The primary intent of radiotherapy is to deliver a measured dose of ionizing radiation to the tumor and draining lymph nodes with the least possible damage to surrounding tissue [1]. Even with vigilant delivery of radiotherapy, acute and delayed skin and mucosal toxicity of some degree is experienced by a substantial proportion of the patients [2].

Acute radiation dermatitis occurs within two to three weeks of starting radiation and can continue beyond three to four weeks post-radiation completion if severe. The appearance time point of acute radiation dermatitis also depends on dose fractionation. The skin changes comprise erythema, edema, burning, pigmentation, dry and moist desquamation, epilation, erosions, ulcers, necrosis, vesiculation, and bullae formations [2,3]. Patients with head and neck cancer receiving radiotherapy are predisposed to develop mucosal toxicity, which is entirely disabling at times. In some patients receiving radiotherapy for head and neck cancer, severe debilitating skin and mucosal toxicity will warrant limiting the dose of radiotherapy, which, in turn, interferes with tumor eradication. Mucosal toxicity further impairs the ability of the patient to eat, deteriorating their already compromised nutritional status, and increases the need for hospitalization and treatment interruption [4]. A systematic review published in 2003, including 23 studies, reported a 16% hospitalization rate and 11% incidence of treatment interruption due to severe mucositis [5]. This coupled with pain, irritation, and dysphagia appreciably worsens the quality of life of patients.

It has been observed that even with equal radiation doses, individual patients respond differently, revealing the importance of additional factors. Risk factors for radiation dermatitis have been studied by researchers and subdivided into patient-related (intrinsic) and treatment-related (extrinsic) factors. Still, investigators lack consensus, with some exhibiting a significant role of age, gender, radiation dose, tissue volume irradiated, comorbidities, smoking, and nutritional status, while others negate it [5]. Thus, there is a need for more studies to delineate these factors in different geographical regions. This study was undertaken to find essential predictors of severe acute radiation-induced skin and mucosal toxicity in patients with head and neck cancer receiving external beam radiotherapy at a single center in the sub-Himalayan region of Uttarakhand, India, so as to guide effective management of at-risk patients.

## Materials and methods

This longitudinal observational study was carried out at the Cancer Research Institute attached to a tertiary care hospital in Dehradun, Uttarakhand, India, over one year from January 2018 to December 2018. The convenience sampling method was used for the enrolment of study subjects. All patients with head and neck cancer aged 18 years or more receiving external beam radiotherapy with a curative intent post-surgery and providing written informed consent were recruited in the study. Patients receiving radiotherapy for palliative intent and those having pre-existing skin diseases were excluded. Institutional Ethics Committee Approval was obtained before the commencement of the study.

A structured case reporting form was used to record data. The dependent variable, the severity of acute radiation-induced skin and the mucosal reaction, was assessed using the Radiation Therapy Oncology Group (RTOG) acute toxicity scoring system (Table 1) [6]. This scoring system is in the form of an ordinal scale ranging from 0 (no change above baseline) to 4 (most severe oral or mucosal reaction). At the start of radiotherapy, patients were seen to collect information regarding age, gender, type and size of cancer, TNM staging, history of smoking, and diabetes. At the end of the radiotherapy, the RTOG score, radiation dose, and concurrent chemotherapy received were noted. In all patients, contouring of target volumes and organs at risk was done with the help of CT simulator unit (SOMATOM Emotion Duo, Siemens Healthcare Pvt. Ltd., Erlangen, Germany) and the treatment planning system (Oncentra MasterPlan, Nucletron Pvt. Ltd., Veenendaal, the Netherlands), and the surface area irradiated was also calculated. The RTOG score was once more noted two weeks after the end of radiotherapy. Scoring was done by a single investigator (R.C.) on all occasions, and appropriate images were captured for the subsequent referral.

**Table 1:** Radiation Therapy Oncology Group (RTOG) acute toxicity scoring system [6].

	Grade 0	Grade 1	Grade 2	Grade 3	Grade 4
Skin	None	Follicular, faint, or dull erythema, epilation, dry desquamation, decreased sweating	Tender or bright erythema, patchy moist desquamation, moderate edema	Confluent, moist desquamation other than skin folds, pitting edema	Ulceration, necrosis, hemorrhage
Mucous membrane	None	Irritation, may include slight pain not requiring analgesic	Patchy mucositis that may produce inflammatory serosanguinous discharge, may include moderate pain requiring analgesic	Confluent fibrinous mucositis, may include severe pain requiring narcotics	Ulceration, necrosis, hemorrhage

The most severe score among the skin or oral mucosa toxicity at the end of radiotherapy or two weeks after radiotherapy was taken as the overall measure of severity. Late toxicities were not recorded. To assess the factors influencing the severity of acute radiation-induced skin and oral mucosa toxicity, RTOG scores of 3 and 4 were considered severe toxicity and scores of 1 and 2 as non-severe toxicity.

The data were entered into Microsoft Excel 2013 (Microsoft, Redmond, WA), and statistical analysis was performed using SPSS Version 22 (IBM Corp., Armonk, NY). Those with severe toxicity were compared with those with non-severe toxicity using the chi-square test for categorical variables and the Student t-test for continuous variables. A p-value of less than 0.05 was considered significant.

## Results

**Figure 1:**
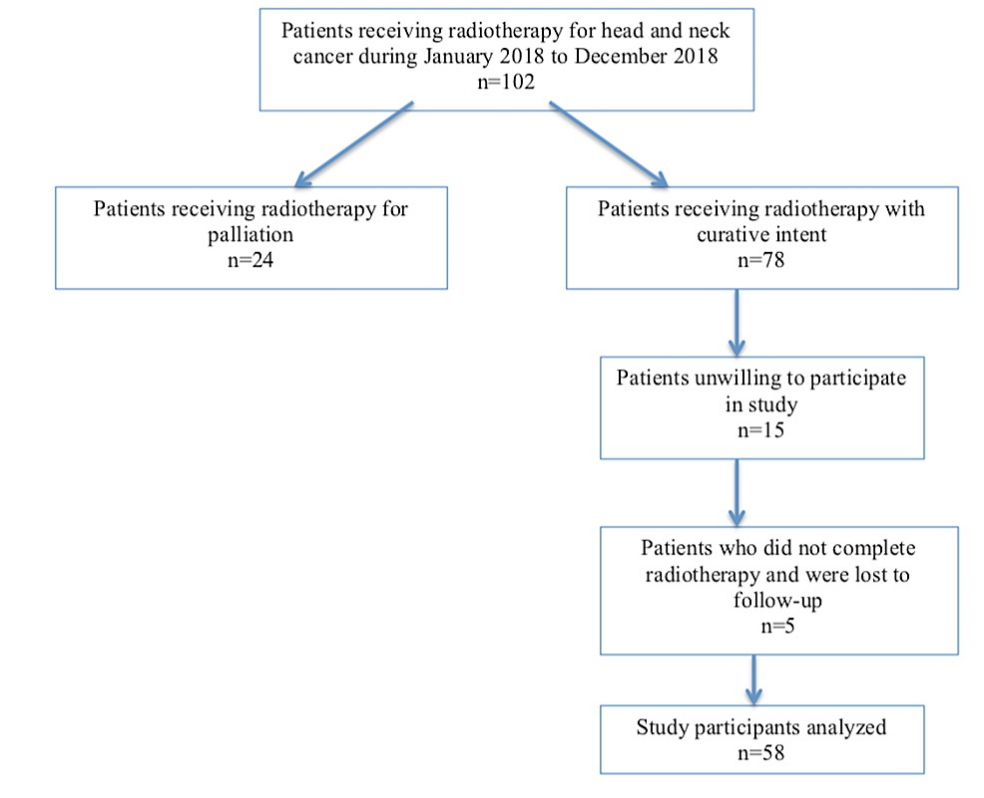
Flowchart of study participants at each stage.

**Figure 2:**
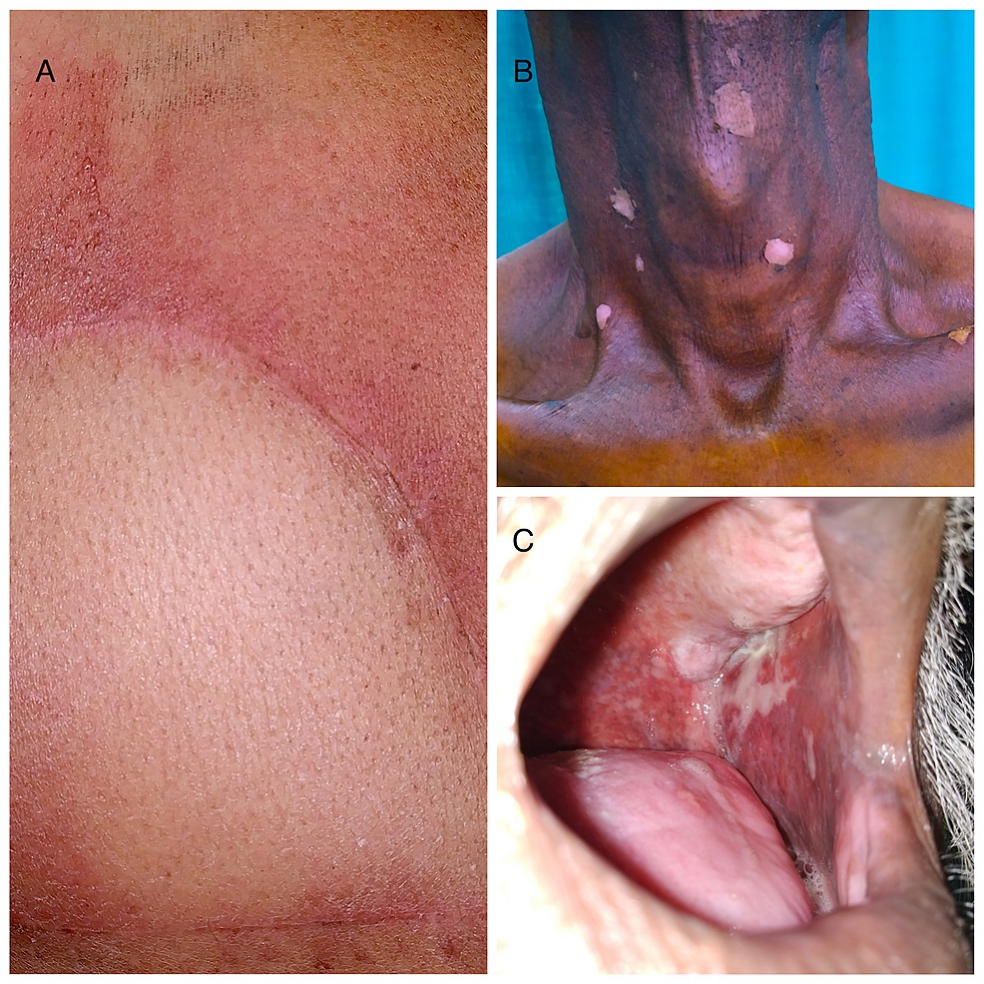
(A) Bright erythema with moderate edema corresponding to RTOG grade 2 cutaneous toxicity. (B) Dull erythema with dry desquamation corresponding to RTOG grade 1 cutaneous toxicity. (C) Patchy mucositis with serosanguinous discharge corresponding to RTOG grade 2 mucosal toxicity. RTOG, Radiation Therapy Oncology Group

A total of 102 patients with head and neck cancer were given radiotherapy during the study period, of which 58 were analyzed (Figure 1). Among the study participants, 48 (82.7%) were men and 10 (17.2%) were women. The mean age of patients was 54.32 years (SD = 13.30; range: 26-85 years). The most common diagnosis was oral/oropharyngeal carcinoma in 43 (74.1%) patients followed by laryngeal/hypo-pharyngeal carcinoma in 12 (20.7%) and nasal cavity/paranasal sinus carcinoma in 3 (5.2%) patients. Majority (79.3%) of patients had moderately differentiated squamous cell carcinoma, and 13.8% and 6.9% had poorly differentiated and well-differentiated types of carcinoma, respectively. TNM stage IV was present in 33 (56.9%) patients, stage III in 20 (34.4%), and stage I/II in 5 (8.6%) patients. Forty-three (68.9%) patients were smokers and five (8.6%) were diabetics. More than half (53.4%) of patients received concurrent chemotherapy. The total dose of radiation received was between 50 and 60 Gray in 52 patients, whereas in six patients, it was either less than 50 Gray or more than 60 Gray. The mean surface area irradiated was 123.30 cm^2^ (SD = 48.53). All patients experienced some extent of acute skin and mucosal toxicity (Figure 2). RTOG skin and mucosa score of 2 was noted in the highest number of patients, seen in 63.8% and 67.2% patients, respectively (Table 2).

**Table 2:** Frequency of proposed predictors and RTOG scores in patients receiving radiotherapy for head and neck cancer (n=58). RTOG, Radiation Therapy Oncology Group

Parameter	n (%) or mean ± SD
Gender	
Male	48 (82.76%)
Female	10 (17.24%)
Age in years	54.32±13.30
Type of squamous cell carcinoma	
Well differentiated	4 (6.9%)
Moderately differentiated	46 (79.3)
Poorly differentiated	8 (13.8%)
Site of carcinoma	
Oral and oropharyngeal	43 (74.1%)
Nasal cavity & paranasal sinuses	3 (5.2%)
Larynx & hypopharynx	12 (20.7%)
Concurrent chemotherapy	31 (53.4%)
Surface area irradiated (cm^2^)	123.30±48.53
Smoker	43 (68.9%)
Diabetic	5 (8.6%)
TNM stage	
I/II	5 (8.6%)
III	20 (34.4%)
IV	33 (56.9%)
RTOG score (skin)	
1	12 (20.7%)
2	34 (63.8%)
3	11 (18.9%)
4	1 (1.7%)
RTOG score (mucosa)	
1	5 (8.6%)
2	39 (67.2%)
3	13 (22.4%)
4	1 (1.7%)

To annul the effect of the dose received, further analysis to study the effect of various factors on the severity of RTOG score was performed in only 52 patients who had received a radiation dose of 50-60 Gray (Table 3). These patients were categorized into two groups: severe toxicity was noted in 15 patients, whereas 37 patients had non-severe toxicity. Comparison of two groups revealed higher age to be associated with severe skin and mucosal toxicity (p = 0.002). Further factors positively associated with severe skin and mucosal toxicity were TNM IV staging (p = 0.023) and concurrent chemotherapy (p = 0.002). However, gender, history of smoking, coexistent diabetes, and surface area irradiated were not statistically associated with severity of skin and mucosal toxicity (p > 0.05) (Table 3).

**Table 3:** Comparison of selected characteristics in patients receiving 50-60 Gray dose of radiation (n = 52) exhibiting severe (RTOG scores of 3 of 4) and non-severe (RTOG scores of 1 and 2) radiation-induced skin and oral mucosal toxicity. RTOG, Radiation Therapy Oncology Group

Parameter	Severe reaction, n = 15	Non-severe reaction, n = 37	P-Value
Age in years (mean± SD)	62.80 ± 9.13	50.81 ± 12.92	0.002
Gender			0.256
Male	73.3%	86.5%	
Female	26.7%	13.5%	
Smoking (%)			0.86
Yes	73.3%	75.6%	
No	26.7%	24.4%	
Diabetic (%)			0.86
Yes	6.7%	5.4%	
No	93.3%	94.6%	
TNM stage (%)			0.023
I/II	0%	13.5%	
III	13.3%	40.6%	
IV	86.7%	45.9%	
Concurrent chemotherapy (%)			0.002
Yes	93.3%	45.9%	
No	6.7%	54.1%	
Surface area irradiated in cm^2^ (mean ± SD)	140.05 ± 61.04	115.12 ± 43.82	0.104

## Discussion

Potential predictors of acute radiation-induced skin and mucosal toxicity have been categorized into treatment-related factors and patient-related factors. Treatment-related factors include large irradiation area, areas with thin epidermis on the face and neck, body folds, bony prominences such as clavicle, incision lines, and peristomal skin, total dose delivered, dose per fraction, and use of bolus [7]. Patient-related factors include advanced age, female sex, surgeries at the site of radiotherapy, smoking, immunocompromised and poor nutritional status, higher body mass index (BMI), fair complexion, genetic factors such as the presence of *IL12RB2* and *ABCA1* genes, history of photosensitivity, diabetes, preexisting connective tissue disorder, and infections such as HIV [8,9]. It is pertinent to study the effects of radiation beam therapy for the early identification to minimize the damage to the skin, which at times can be very disturbing, adding to the sufferings of the patient.

Logically, a higher dose should increase the severity of skin and mucosal toxicity and the same has been well documented [10-12]. However, some researchers have failed to demonstrate this, suggesting that additional factors play a significant role [13,14]. In our study, we have analyzed patients receiving equal dose and fractions of radiotherapy to annul its possible effect, thus helping us pinpoint the additional significant factors. Normal tissue tolerance is thought to decrease with large volumes of tissue exposed. Theoretically, it is easy for small area of skin damage to heal compared to a large area. Thus, surface area irradiated should be positively associated with severity of skin toxicity. In the present study, though the mean area irradiated in patients with severe toxicity was higher (140.05 ± 61.04 cm^2^) compared to those with non-severe toxicity (115.12 ± 43.82 cm^2^), it failed to attain statistical significance (p = 0.104). Previous studies have also disputed this belief shifting the focus more to personal patient factors [10,15].

Concurrent administration of chemotherapy to enhance the effect of radiotherapy is associated with an increase in its toxic side effects on normal cells. A statistically significant relation was established in our study between concurrent chemotherapy and severe toxicity (p = 0.002). This is in concordance with the former studies [1]. Certain agents such as adriamycin, bleomycin, and methotrexate are documented to have an additive or super-additive effect; thus, potential patients need particular attention [1]. Radiation recall dermatitis with chemotherapy drugs, especially taxanes, is well recognized.

Select genetic factors such as gender, coexisting radiosensitive disease (e.g., ataxia telangiectasia), and family history of cancer are anticipated to affect skin toxicity. We were, however, not able to demonstrate a significant association of gender with toxicity, which could be inaccurate considering the disproportionate number of women in the study (male-to-female ratio of 4.8:1). Previous studies have failed to establish an association of severe toxicity with gender, with some substantiating it and others contesting [10,11,16,17].

Personal factors, namely age, comorbidities, smoking, nutritional status, and skin color, have received maximum attention intending to explain the variable effect of radiotherapy. Many of these factors impair wound healing, thus explaining severe toxicity. With advancing age, epidermal turnover decreases, resulting in prolonged healing time. Aging results in atrophy of the epidermis and dermis with a reduction in the capillary network [1]. Higher age was found to be significantly associated with severe skin and mucosal toxicity in our study (p = 0.002), validating the findings of earlier investigators [13,18]. Thus, with advanced age, healing capacity of the skin diminishes. The co-existence of diabetes is expected to increase the severity of skin toxicity by delaying wound healing. We were not able to demonstrate such an association. This could be erroneous as the number of diabetics in our study was limited. Few previous investigators have also reported a non-association [19]. Well-controlled diabetes, which is expected in patients undergoing cancer treatment under constant medical care, does not interfere with skin and mucosal toxicity. Future studies assessing the effect of blood sugar control on toxicity will be able to delineate the precise effect.

Smoking has also emerged as an essential predictor as it impairs tissue oxygenation and thus wound healing. We could not, however, establish such association. Approximately three-fourths of patients in both groups, severe and non-severe toxicity, were smokers (p = 0.86). Positive association of toxicity with the number of cigarettes smoked per day has been demonstrated previously [13].

Higher TNM staging (stage IV) was also significantly associated with severe toxicity (p = 0.023) in the presented study. Meyer et al. compared TNM stage I and II and found that stage II patients had higher skin and mucosal toxicity (OR: 1.91) [17,20]. This could be due to coexistent nutritional deficiencies in patients with advanced disease or a higher tissue volume irradiated.

The primary limitations of our study are a small patient number, disproportionate male-to-female ratio, and less number of people with diabetes, consequently jeopardizing the relevant results. Furthermore, risk analysis based on dose fractionation and the chemotherapeutic agent used could not be performed. Also, the interval to develop cutaneous and mucosal toxicity and time to resolve were not assessed.

## Conclusions

No individual factor is solely responsible for radiation-induced skin and mucosal toxicity. It appears to be an interplay of complex factors behaving differently in individual patients. Thorough knowledge is thus mandatory to minimize severe toxicity and improve the quality of life of cancer patients. Advice to quit smoking, avoid non-necessary photosensitizing drugs, improve the patient's nutritional status, and better control of diabetes mellitus can go a long way in preventing debilitating skin and mucosal toxicity. Older patients with advanced disease requiring concomitant chemotherapy should be appropriately followed to look for early detection of skin and mucosal toxicity, allowing the institution of necessary preventive and therapeutic measures.
